# Mindful eating as the next therapeutic frontier in nutritional psychiatry

**DOI:** 10.3389/fnut.2026.1726847

**Published:** 2026-01-28

**Authors:** Rafael Fernández-Demeneghi, Julieta Sánchez-Bizama, Alma Gabriela Martínez-Moreno, Isidro Vargas-Moreno, Rodrigo Ramirez-Rodriguez, Abraham Puga-Olguín, Erick Yael Fernández-Barradas

**Affiliations:** 1 Instituto de Investigaciones en Comportamiento Alimentario y Nutrición, Universidad de Guadalajara, Ciudad Guzmán, Mexico; 2 Departamento de Ciencias de la Salud y Biomédicas, Facultad de Ciencias de la Salud, Universidad Loyola Andalucía, Sevilla, Spain; 3 Instituto Politécnico Nacional, Ciudad de México, Mexico; 4 Programa Investigadoras e Investigadores por México, SECIHTI-Centro de EcoAlfabetización y Diálogo de Saberes, Universidad Veracruzana, Xalapa, Mexico

**Keywords:** eating behavior, gut-brain axis, mental health, mindful eating, mindfulness, nutritional psychiatry, salutogenesis

## 1 Introduction

The escalating global burden of mental health disorders, with anxiety and depression now among the principal causes of disability affecting over one in eight individuals, constitutes a critical public health crisis (1). Although these conditions emerge from a complex interplay of genetic, psychosocial, and environmental factors, there is increasing consensus that lifestyle interventions—especially dietary patterns—represent modifiable targets for both prevention and treatment (2). Within this context, Nutritional Psychiatry has emerged as an essential interdisciplinary field, elucidating the bidirectional pathways through which diet shapes brain function and emotional wellbeing. Still, contemporary therapeutic frameworks must evolve beyond a reductionist focus on isolated nutrients to address the fundamental determinants of mental health—*how, when*, and *why* we eat (3–5). Eating behaviors involve complex emotional, cognitive, and bodily processes influencing diet, stress, and mental health, but are often overlooked in clinical research (6, 7).

Mindfulness and mindful eating offer behavioral strategies uniquely positioned to address this gap by cultivating present-moment awareness and attunement to internal and external cues. Accumulating neuroscientific evidence suggests that mindfulness-based interventions recalibrate reward processing, enhance neurocognitive flexibility, and facilitate stress regulation (8–10). Notably, mindful eating has demonstrated efficacy in improving eating behaviors related to overweight and obesity, conditions closely linked with mental health disorders, by reducing emotional and binge eating and improving self-regulation (5, 6). Neuroimaging data indicate that mindful eating can modulate the salience of food cues, dampen activity in the midbrain reward pathway, and strengthen prefrontal emotion regulation networks (9, 11).

Despite promising advances, this field faces challenges including conceptual ambiguity, methodological variability, and a scarcity of long-term mechanistic studies (12, 13). This Opinion advocates that mindfulness and mindful eating can directly engage core biobehavioral mechanisms implicated in psychiatric disorders—including reward sensitivity, hedonic hunger, the gut-brain axis, and neuroplasticity. Framed within a salutogenic model prioritizing health promotion, mindful eating emerges as an accessible, low-risk approach that fosters psychological resilience and enables a paradigm shift toward holistic, personalized, and preventive mental health care.

Adopting an integrative perspective that extends beyond isolated nutrients to encompass how, why, and how much we eat, we define Mindful Eating as a multidimensional biobehavioral framework rather than a mere modification of ingestion speed. It is conceptualized as the active integration of cognitive, emotional, and interoceptive domains, characterized by non-judgmental attentiveness to the complete sensory experience and internal physiological cues of hunger and satiety. Operationally, this mechanism serves to disrupt behavioral automaticity and decouple food intake from emotional reactivity, thereby realigning dietary decision-making with metabolic homeostasis rather than hedonic reward processing.

## 2 Beyond nutrients: eating behavior as a therapeutic target in nutritional psychiatry

The traditional focus in nutritional psychiatry on isolated nutrients has inadvertently fostered a reductionist perspective, overlooking the complex biopsychosocial nature of eating behavior (2, 14). Eating transcends mere biochemical ingestion; it is an intricate behavior influenced by emotional, cognitive, and social contexts that together shape dietary adherence and clinical outcomes (4, 15). Overlooking these behavioral dimensions may limit the effectiveness of nutrient-centered interventions (16).

A critical flaw in many current dietary interventions is the implicit assumption of rational, linear adherence to guidelines, which overlooks how emotional states, habitual cues, and cultural scripts powerfully shape actual eating behavior. Mindful eating directly addresses this gap. By fostering heightened awareness and self-regulation, it shows preliminary promise in decoupling eating from hedonic and emotional drivers and in enhancing dietary consistency (5–7). However, well-powered, longitudinal trials are needed to elucidate its long-term impact on energy balance and metabolic health.

For nutritional psychiatry to realize its full potential, it must treat eating behavior not merely as a confounder but as a primary therapeutic target. Cultivating a mindful, positive relationship with food could empower individuals to adopt and maintain nutrient-rich diets, maximizing the preventive and therapeutic benefits of nutrition for mental health.

## 3 Mindfulness and mindful eating: mechanisms relevant to mental health

**Figure 1:**
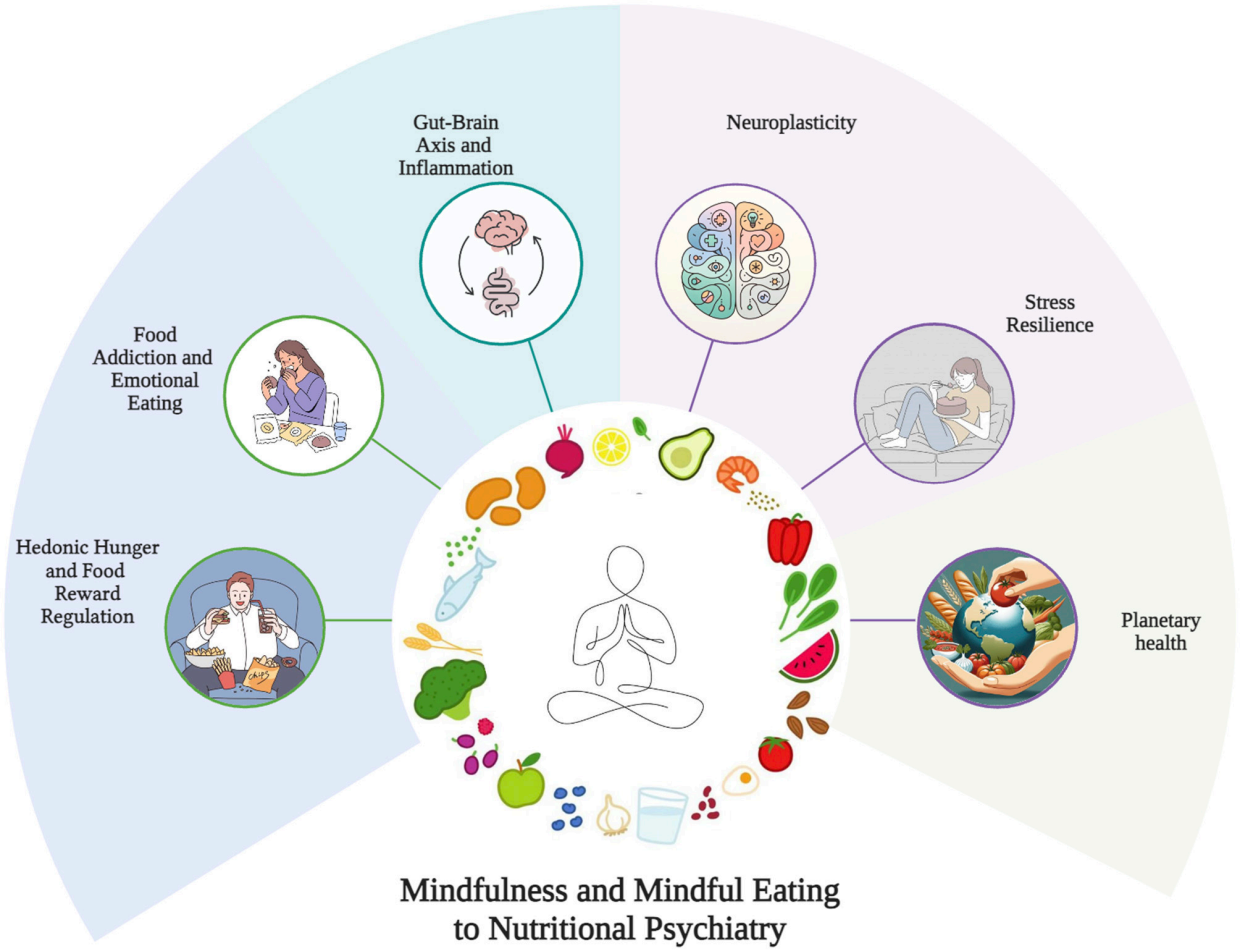
Core biobehavioral mechanisms of mindfulness and mindful eating in nutritional psychiatry. An integrative mechanistic framework illustrating how mindful eating promotes mental health. This proposed model integrates the neurobiological modulation of reward and executive control circuits; the physiological regulation of the gut-brain and stress axes; and the cognitive enhancement of self-regulatory capacities. These synergistic pathways are posited to converge, thereby underpinning psychological resilience. Innovatively, the framework extends to a socio-ecological dimension, suggesting that the cultivation of intentional eating bridges individual wellbeing with the broader principles of planetary health.

Mindfulness-based interventions engage complex neurobiological and behavioral pathways essential to mental health, making them promising tools in nutritional psychiatry (5, 15). These approaches recalibrate maladaptive responses to hyperpalatable food cues, promote behavioral flexibility, and engage brain circuits involved in reward and stress regulation, thus offering pathways to reshape eating patterns and enhance psychological wellbeing (7, 9) (see Figure 1).

### 3.1 Hedonic hunger and food reward regulation

Hedonic hunger refers to eating for pleasure rather than metabolic need, mediated by dopamine-driven reward pathways in the striatum and midbrain (17). Mindful eating appears to attenuate these hypersensitive circuits by encouraging nonjudgmental observation of cravings, decoupling conditioned food cues from compulsive eating, and modulating reward signaling (9, 11). Neuroimaging shows reduced mesolimbic reward activation and improved executive control, consistent with enhanced top-down regulation (9, 10).

### 3.2 Food addiction and emotional eating

Mindful eating also holds significant promise for mitigating compulsive and addiction-like eating patterns, which share neurobiological substrates with substance use disorders. By fostering cognitive decentering—the ability to observe thoughts and urges as transient mental events—mindfulness-based approaches disrupt the cycle of emotional eating and impulsive consumption, thereby facilitating sustainable behavioral change (5–7).

### 3.3 Gut-brain axis and inflammation

The influence of mindful eating extends to the gut-brain axis. While precise mechanisms remain under investigation, evidence suggests this relationship is mediated largely by autonomic modulation via the vagus nerve (18, 19). Chronic stress and distracted eating heighten sympathetic tone, which can increase intestinal permeability and alter gut motility. Conversely, by fostering a parasympathetic state during ingestion, mindful eating promotes vagal tone, creating physiological conditions that favor more diverse and resilient gut microbiota profiles. This includes an increased abundance of taxa such as *Bacteroides* and *Lactobacilli*, which produce anti-inflammatory short-chain fatty acids and support gut barrier integrity (20). Concurrently, mindfulness practice downregulates the hypothalamic-pituitary-adrenal (HPA) axis, reducing chronic stress and lowering circulating proinflammatory cytokines that are strongly implicated in the neuroinflammation characteristic of mood disorders (21, 22). These interconnected pathways position mindful eating as a synergistic intervention that modulates both systemic and neural health. While these associations are compelling, current evidence is mainly correlational, and the direction of causality remains to be firmly established. Longitudinal studies tracking changes in both microbiota composition and psychological states following mindful eating interventions are essential to disentangle this complex bidirectional relationship.

### 3.4 Neuroplasticity and stress resilience

Mindfulness and mindful eating may drive adaptive neuroplastic changes that underpin lasting mental resilience, though the precise mechanisms remain an active area of investigation (23, 24). Structural MRI studies consistently report increased gray matter density and cortical thickness in brain regions critical for executive function and emotional regulation, including the prefrontal cortex, anterior cingulate cortex, and insula (24–26). These anatomical changes are thought to reflect enhanced dendritic arborization and synaptogenesis.

Functionally, mindfulness training systematically modulates key large-scale brain networks. It has been shown to reduce hyperactivity and connectivity within the default mode network (DMN), a system linked to mind-wandering and rumination, thereby mitigating maladaptive self-referential thought (27, 28). Simultaneously, it strengthens connectivity within the central executive and salience networks, supporting enhanced attentional control, cognitive flexibility, and emotional regulation—all core components of stress resilience (29, 30). At a molecular level, preliminary evidence links mindfulness with increased expression of brain-derived neurotrophic factor (BDNF), a key molecule for synaptic plasticity and neurogenesis (23, 31).

Collectively, these neurobiological adaptations—from cortical structure to network dynamics and molecular signaling—provide a compelling mechanistic basis for how mindfulness and mindful eating can remodel neural architecture to support healthier eating behaviors and long-term mental health (32). It is important to note that much of the foundational neuroimaging evidence for these neuroplastic changes derives from studies on general mindfulness-based interventions rather than mindful eating protocols specifically. While mindful eating shares foundational elements with general mindfulness practice, specific evidence on its impact on neuroplasticity remains limited and extrapolated mainly from broader interventions.

### 3.5 Planetary health diet and mental wellbeing

Exposure to green spaces improves mental health by reducing stress and depression while encouraging physical activity and social connection. This wellbeing boost complements adherence to sustainable diets like the EAT-Lancet Planetary Health Diet, which emphasizes plant-based foods and limits red meat, reducing depression risk and mortality (33–35). Mindful eating further supports intentional, health- and planet-conscious choices, strengthening the link between mental health and environmental sustainability (7, 36). Together, these factors form a holistic approach to planetary mental health. While specific dietary compositions may vary by clinical or cultural needs, the attentional quality of Mindful Eating serves as a foundational skill for sustainable behavior change.

## 4 Salutogenesis and mental health: a theoretical integration

Salutogenesis offers a transformative framework for mental health by focusing on factors that actively promote and sustain wellbeing (37). Central to this model is the sense of coherence, encompassing comprehensibility, manageability, and meaningfulness (38). Mindful eating can operationalize these elements by fostering awareness of internal cues, enhancing coping with triggers, and aligning food choices with personal values. This approach transcends prescriptive dietary advice, empowering individuals to develop their own General Resistance Resources (GRRs) and navigate complex food environments with resilience (39–41).

As a scalable, low-cost intervention, mindful eating is well-suited to diverse public health settings. School-based programs employing mindful eating demonstrate promising effects in cultivating healthier relationships with food and promoting psychological resilience (42). Such initiatives exemplify salutogenic strategies that expand both individual and collective resources, fostering self-awareness, agency, and sustainability in mental health care (43)—contributing meaningfully even to planetary health goals.

## 5 Discussion, limitations, and future directions

Mindfulness and mindful eating are increasingly recognized as behaviorally grounded strategies with neurobiological relevance to mental health. By enhancing interoceptive awareness, fostering cognitive flexibility, and reducing emotionally driven and dysregulated eating patterns, these practices target core mechanisms implicated in psychiatric conditions—including dysregulated reward processing, chronic stress, neuroinflammation, and impaired self-regulation (5, 6, 8, 44). Despite growing interest, several conceptual and methodological limitations continue to impede their integration into clinical and public health frameworks.

A persistent challenge lies in the inconsistent operationalization of mindfulness and mindful eating. As emphasized by Mantzios, definitional variability undermines comparability across studies and obscures which elements are truly therapeutic (13). Moreover, mindful eating interacts with co-factors such as self-compassion, attentional control, and emotional context—dimensions that are rarely systematically accounted for (45, 46). There is an urgent need for standardized reporting guidelines and multidimensional frameworks that reflect this complexity while improving reproducibility.

Methodological heterogeneity also extends to intervention protocols, which vary widely in duration, delivery format (digital vs. in-person), intensity, facilitator training, and cultural contextualization. This variability complicates the synthesis of findings and the identification of active ingredients. Notably, mindfulness-based approaches may not suit all populations equally. Individuals with trauma histories, severe eating disorders, or cognitive vulnerabilities may require trauma-informed adaptations, increased clinician support, and flexible engagement strategies (6, 8).

Recent findings also point to underexplored but compelling mechanisms. Mindful eating has been linked to increased behavioral flexibility, improved reversal learning, and even enhanced alignment with sustainable food choices and pro-environmental values—suggesting a broader potential for systemic impact (10, 36). Integrating these findings into future trial designs could inform interventions that simultaneously address psychological, physiological, and ecological wellbeing.

However, while this synergy with sustainability is promising, Mindful Eating should fundamentally be understood as a diet-agnostic intervention. Its core mechanisms—specifically interoceptive awareness and impulse regulation—are equally relevant across diverse nutritional contexts and are not contingent upon specific macronutrient profiles. This distinction is particularly relevant for metabolic psychiatry, where restrictive interventions such as ketogenic diets are increasingly utilized for symptom management in conditions like bipolar disorder and schizophrenia (47). Furthermore, the universality of mindful eating extends to cultural contexts with traditionally high animal-product consumption, such as circumpolar populations. In these settings, the therapeutic utility of Mindful Eating lies not in enforcing a plant-forward standard, but in optimizing the bio-behavioral relationship with food. By enhancing sensitivity to satiety signals, it supports metabolic regulation regardless of whether the dietary pattern is plant-based or animal-based (48, 49).

To advance the field, a focused and interdisciplinary research agenda is essential. Key priorities include:

- Large-sample, longitudinal randomized controlled trials (RCTs) with active comparators, longer follow-up, and standardized outcome measures in clinically diagnosed populations.
- Mechanistic studies employing objective biomarkers, including functional and structural neuroimaging, cortisol, inflammatory cytokines, gut microbiota composition, and brain-derived neurotrophic factor, to identify mediators of effect and individual predictors of response.
- Hybrid trials combining mindful eating with dietary interventions—ranging from the Mediterranean, Milpa diet or functional food like berries (50) examining whether behavioral self-regulation enhances dietary adherence, bioavailability, or synergistic therapeutic outcomes.
- Component and mediation analyses to isolate active ingredients (e.g., formal meditation vs. informal awareness vs. interoceptive training) and explore dose–response effects.
- Implementation science research, assessing feasibility, cost-effectiveness, digital scalability, and cultural adaptability across varied settings—including schools, primary care, and public health campaigns (51).
- Personalization strategies, informed by baseline characteristics (e.g., interoceptive accuracy, trauma history, motivational readiness), to tailor interventions and enhance engagement and efficacy.

Beyond clinical application, mindful eating aligns with a salutogenic model of mental health promotion. By cultivating attentional stability, emotional regulation, and embodied awareness, it empowers individuals to develop more sustainable, intentional relationships with food—supporting resilience across diverse populations, ages, and cultural contexts. Its low risk, adaptability, and potential scalability position it as a compelling adjunct within lifestyle-based mental health frameworks.

While mindful eating is not a standalone treatment for psychiatric disorders, it represents a biologically plausible and theoretically robust tool that bridges disciplines—linking neuroscience, nutritional science, and behavioral psychology. Future work must prioritize methodological rigor, multidimensional measurement, and culturally informed design to realize its transformative potential in nutritional psychiatry fully.
